# Black Cohosh Hepatic Safety: Follow-Up of 107 Patients Consuming a Special *Cimicifuga racemosa rhizome* Herbal Extract and Review of Literature

**DOI:** 10.1093/ecam/nen009

**Published:** 2010-12-23

**Authors:** Fabio Firenzuoli, Luigi Gori, Paolo Roberti di Sarsina

**Affiliations:** ^1^Clinical Center of Natural Medicine, S. Giuseppe Hospital, Empoli, Italy; ^2^Italian High Council of Health, Ministry of Health, Rome, Italy

## Abstract

European Medicines Agency (EMEA) and the Committee on Herbal Medicinal Products (HMPC) on July 2006 have released an alert to get European sanitary authorities aware of 42 cases of suspected hepatotoxic reactions in patients consuming *Cimicifuga racemosa rhizome*. In the public statement EMEA itself considered reliable as hepatotoxic reactions only four cases, on the base of RUCAM score: two were considered possible and two probable. Lacking in almost all of them a precise description of cases, especially a botanical-chemical analysis of the suspected substance, we think there is no real proof of supposed *C. racemosa rhizome* hepatotoxicity. In our department we administer from about 10 years *C. racemosa* as special herbal dry extract as single substance or mixed with other medicinal plants at the dose of 500–1000 mg daily, for treatment of menopause related disorders without any reported adverse effect. After EMEA's official signal we have contacted all our patients using a *C. racemosa rhizome* herbal extract continuously from more than 12 months to verify possible hepatotoxic effects. We followed-up 107 women, and asked them by telephone (33/107) and/or after anamnesis and clinical examination (74/107) to undergo a blood sample examination. In all the patients there was no sign of hepatic disease, or worsening of already altered but stable parameters. We think on the base of these data and current literature *C. racemosa rhizome* extract should not be considered a potential hepatotoxic substance.

## 1. Introduction

Currently hormone replacement therapy (HRT) is commonly used to reverse symptoms and diseases associated with falling oestrogen and progesterone levels. However HRT is associated with adverse effects and an increased risk of breast cancer [1]. Many women therefore take a dislike to HRT and choose to employ herbal remedies for menopausal symptoms, one of most used worldwide is Black Cohosh.

**Figure 1:**
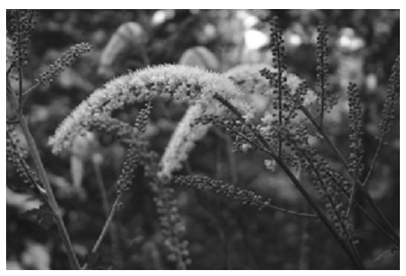
Cimicifuga racemosa Nuttal.

*Cimicifuga racemosa* Nuttal (syn. *Actaea racemosa* L) common name Black Cohosh (Ranunculaceae; Figure 1), is an herbaceous perennial plant of North America. It is a coarse, perennial, woodland herb with large compounds leaves, and a thick, knotted, rhizome system [2]. Traditionally it was used by Native Americans (Penobscot, Winnebago and Dakota) [3] for the treatment of coughs, colds, constipation, fatigue and rheumatism and to stimulate lactation. Since 1832 a hydroalcoholic extract was described as treatment for pain and inflammation in endometriosis and dysmenorrhea [3]; and a fluid extract was listed in the US National Formulary from 1840 until 1946 [4]; and was a major constituent of the once popular patent medicine “Lydia Pinkham's Vegetable Compound” used for treatment of “painful complaints and weakness” in females. Actually, *C. racemosa* is widely employed in various pharmaceutical–industrial preparations often mixed with other medicinal plants to alleviate menopausal and post-menopausal symptoms, including hot flushes, profuse sweating, sleep disturbances, anxiety and depression.

Safety data from post-marketing surveillance study have generally found very few serious adverse events, nevertheless the lack of placebo or positive control arms in main studies, the lack of serious adverse events suggests that *C. racemosa* has a very good safety profile [4] as confirmed in a review of more than 3800 climateric women [5].

Recently the European Medicines Agency (EMEA) and the Committee on Herbal Medicinal Products (HMPC) on July 2006 have released a public statement [6], although lacking of many data on patients and herbal extracts, to get European sanitary authorities aware of 42 (34 directly reported from European national competent authorities and 8 published) cases of suspected hepatotoxic reactions in patients, probably all women and for treatment of menopause related disorders, consuming *C. racemosa* often mixed with other medicinal plants. Following the review of available data, the statement considered real a potential connection between herbal medicinal products containing *C. racemosa* and human hepatotoxic reaction. Nevertheless this highly authoritative review of such a number of cases, in the summary EMEA states that “overall, all discussed cases of literature and pharmacovigilance reports are poorly documented. In many cases there is not even information about the time frame of treatment with *Cimicifuga* containing products or about the relation to the onset of reaction available. Therefore, they are insufficiently documented according to RUCAM score. The Non-EU cases are not validated by a health care professional but reported by patients” [6]. Because *C. racemosa* extracts are sold as OTC integrators, Italian sanitary authorities decided a precautionary withdrawal from the national market, that later has been reversed, and stringent label warnings have been introduced for *C. racemosa* extracts in United Kingdom. So actually there are some concerns about *C. racemosa* extracts safety especially in patients suffering of liver disease.

## 2. Methods

### 2.1. Participants

**Figure 2:**
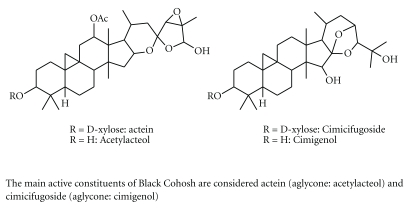
The main constituents of Black Cohosh.

In our department we administer *C. racemosa rhizome* extract regularly for treatment of menopause related complaints, from about 10 years to 798 patients as only substance or mixed with other medicinal plants like *Glycine max* isoflavones, *Trifolium pratense* and. *Medicago sativa*, at the dose of 500 or 1000 mg daily as dry extract, standardized and titrated in 2.5% of actein, a triterpene glyocoside (Figure 2), for treatment of menopause related disorders like anxiety, depression, flashes and myalgia. We regularly exclude from the treatment patients affected by cancer of sexual organs: breast, ovaries, uterus and hypophysis, unless already treated with conventional therapy from more than 7 years and considered recovered. In our experience we had no report of adverse reaction to *C. racemosa* extracts, both as single prescription either mixed with other medicinal plants extracts, but minor complaints.

After EMEA's official statement indicating a possible risk of hepatotoxicity in patients assuming extract of *C. racemosa*, we have contacted all our available patients consuming the extract continuously from at least 12 months.

So we considered 158 patients eligible, for control of a possible hepatotoxic adverse reaction to *C. racemosa* herbal extract to whom it was prescribed following our patterns. Of our contact list 107 climateric women were followed-up by telephone or direct clinical examination in our department.

### 2.2. Study Protocol

Baseline characteristics of study participants are available in Table 1. They were an heterogeneous group of patients in different phases of menopause and most of them asked a complementary treatment for menopause related anxiety and depression because of unsatisfied or fear of synthetic drugs. Most of them were in good health, but complaints referred to menopause. Only five patients were suffering of chronic hepatobiliary disease (four patients suffering of benign hepatic disease and one chronic toxic hepatitis). We excluded from follow-up patients who had suspended the treatment for more than 15 days, also non-consecutive, or had spontaneously reduced the dose prescribed.

We followed-up 107 women asking them by telephone (33/107) and/or after clinical examination (74/107), to undergo a blood sample examination to control main laboratory parameters to evaluate liver function.

As shown in Table 2 patients underwent a blood sample examination to evaluate: total leukocyte count, hepatic transaminases, *γ*-glutamiltranspeptidase, alkaline phospahatase, albumin, coagulation and total bilirubin. By telephone the patients were questioned if they were still regularly consuming the herbal extract and they were asked some question on the base of the same check-list used during clinical examination in hospital.

## 3. Results

In all the patients (comprehending four patients suffering of benign hepatic disease and one suffering of chronic toxic hepatitis) there were no sign of hepatic disease neither alteration of plasma hepatic parameters, or worsening of already altered but stable parameters. Only nine patients were suffering of minor transient complaints (see Figure 2) and after 1 month underwent by telephone a new follow-up with a blood sample control that was negative for any disease.

To our patients we administered very high doses respect to median dose used in clinical trials (median range 40–80 mg daily) [7, 8], but after 12 months of regular use there were no laboratory data, neither clinical signs, referring to a possible hepatic adverse reaction.

Few patients (9/107) were suffering of minor complaints, like fatigue, aspecific abdominal pain and paresthesias, (Table 2), controlled again after 1 month did not show any sign of liver disease.

## 4. Discussion

### 4.1. Hepatotoxic Drug Reactions

Between 0.1 and 3% of all hospital admissions are related to drug toxicity, but it is suspected that many cases are not recognized. Since liver is central to the metabolic disposition of virtually all drugs, drug-induced hepatic injury is a potential complication of nearly every medication. Fortunately adverse hepatic drug reactions are relatively uncommon in comparison with the number of drug prescriptions. In majority of the cases, hepatic drug reactions present as acute hepatitis, which is usually reversible and relatively benign; however, the spectrum of liver disease may range from mild biochemical abnormalities to acute hepatic failure.

When a drug is found to cause even rare hepatotoxicity but is used by millions, it may disproportionally be removed from the market, although this substance is a real danger only for few patients. And should be stressed that to incriminate any drug in an episode of liver dysfunction is a difficult step-by-step process that requires: high degree of suspicion, compatible chronology, awareness of its hepatotoxic potential, competent exclusion of alternative causes of liver damage and the ability to detect the presence of subtle data that favors a toxic etiology [9].

It is very important in the step-by-step process to rule out other causes of liver injury including hepatic infections, alcoholic and autoimmune hepatitis, biliary tract disorders and hemodynamic based diseases [10]. Finally genetic and metabolic disorders may produce liver injury such as hemochromatosis and Wilson's disease. Liver injury in the absence of other known causes may be drug-related and requires additional information, such as that obtained through a careful drug history, in relation to the onset of injury [10].

In respect to the hepatotoxic potential of screened drugs, their potential for causing liver damage is not the same, and almost all marketed medications have been incriminated in incidences of hepatotoxicity [9, 11]; probably because the most important factor for hepatotoxicity is genetic variability [12]. Genetic polymorphisms have a strong influence on drug metabolism and may increase the risk of hepatotoxicity [13].

The main causative group of drugs, in a large cohort of hepatotoxicity cases collected in the Spanish registry, were antibiotics followed by non-steroidal anti-inflammatory drugs [14]; and statins have been shown to cause elevations of aminotrasferase levels and severe liver injury in animals; while in humans such elevations are common but rarely if ever, lead to clinically significant hepatotoxicity [15]. All drugs that are well known hepatotoxic, and well stable in the market, thanks to their therapeutic advantages, as such as paracetamol, one of main causes worldwide of liver transplant, nevertheless sold over-the-counter in many Western countries.

### 4.2. Safety of Black Cohosh in Experimental Trials

A study *in vitro* on HepG2 cells and *in vivo* on rats to evaluate potential hepatotoxicity of *C. racemosa*; showed cytotoxic reactions *in vivo* at concentrations of 75 *μ*g ml^−1^; and significant initial mithocondrial swelling in rats fed with 100 and 300 mg kg^−1^ daily of *C. racemosa* extract, while clear microvesicular steatosis of the hepatocytes and fragmentation of the rough endoplasmic reticulum at 1000 mg kg^−1^ daily [16]. The Authors conclude that toxicity is not clinically relevant for most patients but may become important in patients with underlying risk factors [16], as like as for any drug; moreover a dose of 100 mg kg^−1^ is very high and corresponds in a woman of 50 kg at 5 g of extract daily.

Natural and synthetic estrogens are known to alter hepatobiliary physiology, and certain genetically susceptible women become icteric during pregnancy, when high serum oestrogen levels are present. Although not all the mechanisms responsible for hepatic injury produced by estrogens are known, it is consistently reported that Black Cohosh extracts and isolated compounds do not posses estrogenic activity, regardless of source, extraction procedure, dose or length of exposure [17].

Chronic toxicity was not observed in rats at a dose of 500 mg kg^−1^ per day for 27 weeks or in dogs at 400 mg kg^−1^ per day for 26 weeks [18]. Furthermore, a 40% 2-propanol extract of Black Cohosh was negative in the Ames test [18]. In mice, the LD50 of a Black Cohosh extract was 7.7 g kg^−1^ for intragastric administration and 1.1 g kg^−1^ for intravenous administration [18].

### 4.3. Safety of Black Cohosh in Humans

In clinical trials only minor adverse side effects have been reported, including nausea, vomiting, head-aches and dizziness: a review of eight clinical trials concluded that extracts of the rhizome of *C. racemosa* might be a safe alternative for women seeking alternative estrogen replacement therapy [19].

Black Cohosh also contains several catechols, such as caffeic acid, piscidic acid and fukiic acid esters that exhibit some antioxidant properties, including fukinolic acid, cimicifugic acid A and cimicifugic acid B [20]. Such catechols could be of significant concern in toxicology because of the possibility that they could be activated, either metabolically or chemically, to electrophilic quinones. The potential of such quinones to cause toxicity and carcinogenesis is well documented, and can occur via arylation of cellular proteins and DNA or redox cycling leading to the formation of reactive oxygen species such as the hydroxyl radical [21]. Nevertheless has been shown in six perimenopausal women after administration of a single dose of either 32, 64 or 128 mg of *C. racemosa* that no corresponding mercapturic acids were found in the urine [22]. In a previous study, potential toxicity was suspected because catechols from Black Cohosh are activated to quinoid metabolites, but catechols are not absorbed across the bowel [23].

In a recent trial on 351 randomized women, placebo controlled, Black Cohosh both used as single substance and mixed in multi herbs remedies after 12 month of treatment, did not show any effect on lipids, glucose, insulin and fibrinogen [24].

Instead an important issue about safety is probably the interaction with synthetic drugs because of the interference with the metabolic pathway of cytochrome P4503A4 [25], a potential cause of important adverse reactions, especially in patients assuming multi drug regimen therapies as confirmed by a recent work in which ethanol and isopropanol extract induced inhibition of CYP1A2, 2C9, 2D6, 3A4.

To be added a case of cutaneous pseudolymphoma in a patient assuming a commercial extract of *C. racemosa* has been reported recently [26]; and a case of muscle damage with asthenia, high levels of creatine phosphokinase and lactate dehydrogenase following assumption of a dietary supplement derived from *C. racemosa* has also been reported [27].

### 4.4. The EMEA and HMPC Signal of Hepatic Toxicity

The EMEA and the HMPC have been made aware of a number of case reports of hepatotoxicity (liver injuries) in patients using *C. racemosa rhizome*. Following review of all available data, the HMPC considered that there is a potential connection between herbal medicinal products containing *C. racemosa rhizome* and hepatotoxicity on the base of 42 case reports of hepatotoxicity, collected from European National Competent Authorities (34 cases) as well as literature case reports (8 cases). Of these, only 16 cases were considered sufficiently documented to allow the Committee to assess if use of *C. racemosa rhizome* could be linked to the liver injuries. As a result of the assessment, five cases were excluded and seven cases were considered unlikely to be related. In the remaining only four cases (two autoimmune hepatitis, one hepatocellular liver injury and one fulminant hepatic failure), there was a temporal association.

So there are very few cases well documented and few data available, for a concrete decision about its suspected hepatotoxicity; and in many reports *C. racemosa* extracts were mixed with many other substances so that was impossible to get any reliable reference to the assumption of *C. racemosa*.

Besides, in the best four cases is not possible to evidence a clear hepatotoxic relationship with the consumption of *C. racemosa* extract:

- In case 28, as numbered in the EMEA statement, (RUCAM Score 4) the only collected from European national competent authorities, the adverse reaction is considered possible but information on differential diagnostic assessment were not available. The patient was assuming 80 mg of an unknown type of extract daily, for an unknown period and without differential diagnostic available.
- In this published case report (RUCAM score 3) [28] the patient was a 54-year-old woman suffering of hypothyroidism, fibromyalgia, osteoarthritis and depression that had taken 1000 mg of Black Cohosh (no description of extract or crude drug) daily for several months. The patient was on fluoxetine, propoxyphene and paracetamol (a well known hepatotoxic substance) concomitantly and drinking two glasses of wine in the evening. In the statement it is written “that since there is no further information on the herbal preparation, the case report is not of great relevance in the assessment of cimicifuga-related hepatotoxicity” [6].
- A 57-year-old multimorbid African American woman [29] (RUCAM Score 7) with a history of polymyositis had developed an autoimmune hepatitis about 3 weeks after the first intake of *C. racemosae rhizoma* (brand and dose unknown; no information if it was a combination product). After cessation of the drug and starting a therapy with steroids and azathioprine, the patient recovered. The autoimmune hepatitis could have been as well a manifestation of a multisystem autoimmune disease. According to the draft recommendations of the Scientific Advisory Panel Subgroups on Hepatotoxicity, the case could be classified as idiosyncratic liver necrosis.

The causal relationship to Black Cohosh in the present case was first assessed as “unclassifiable” as information provided concerning the reaction was regarded as incomplete and contradictory, while the follow-up publication includes more (although to some extent contradictory) therapy information.

- This a well-documented case (RUCAM Score 6) [30]. The admitted amount was 500 mg drug per day. The reaction was described as hepatocellular, other causes for acute hepatitis were excluded. The provisional clinical diagnosis was autoimmune hepatitis. Therapy was started with 60 mg prednisone/day for 5 weeks. Liver enzymes improved, but due to worsening coagulopathy and encephalopathy the patient underwent an orthotopic liver transplantation. In the explanted organ, histologically features of acute hepatitis, fibrous linkage of portal tracts and cholestasis were seen after 5 weeks on corticosteroids. Nevertheless in this case too, the exact content of the remedy based on Black Cohosh is not known: was it an extract or crude drug? Did it contain other toxic substances? Heavy metals? Later on nevertheless Levitsky et al. [30] reported that the patient did not drink, use drugs or take any other medication; but in a US District Court proceeding the woman testified that she regularly drank wine, and used both prescribed and non-prescribed pharmaceuticals [31].

### 4.5. The Problematics of Herb ADRs

We would like to remark that it is very important before an official statement about any adverse reaction referred to an herb based product to know the brand, dose of substance assumed, type of extract, content of possible contaminants (wrong plants, pesticides, heavy metals and aflatoxins) [32, 33]. When needed to establish a connection between herb consumption and an adverse reaction, especially if liver related, the same rules of conventional drugs can be followed [32] only if the case report deal with a single substance of a medicinal plant; while if it is a multiherb preparation a direct toxic connection with only one herb cannot be correctly established, unless it is a well known hepatotoxic substance for example, *Teucrium chamaedrys*; or it is contained in a very high ratio respect to other herbs. To date in our opinion paradoxically the EMEA statement could be regarded as the proof the risk of Black Cohosh hepatotoxicity is scarce, because actually there is not any full proved case of hepatotoxicity reported in front of millions of doses used yearly in the world (about 1 500 000 women are using *C. racemosae* extracts only in Europe) [34]; and its safety was already been sufficiently established by the fact that in over 3800 participants in clinical trials there were no hepatotoxic reactions reported [5].

In our series of patients assuming daily a very high dose of extract (much more than 40–80 mg used in most clinical trials and usually recommended) [7, 8, 23] for at least 12 months we did not find any clinical or laboratory proof of liver disease; but the five already suffering of chronic liver disease, that maintained stable clinical and laboratory parameters (Table 3).

In our series we had a relative low number of patients (32/107) assuming contemporary synthetic drugs to exclude reliably an interference on hepatic metabolism, although they did not report any significant side effect.

## 5. Conclusions

A reporting rate higher than the background rate is taken as a “signal” of a possible causal relationship between the drug and the event [35], but in the EMEA statement the reported cases are so faintly circumstantiated that we think there is no evidence for an official signal. On the base of our data (although a small series of patients, but assuming high doses of *C. racemosae* extracts) and current literature, we think *C. racemosa rhizome* herbal extract should be considered safe concerning liver toxicity.

## Figures and Tables

**Table 1:** Baseline characteristics of study participants.

Characteristics	*N* = 107 (%)
Age	48 (SD 4.24)
Menopause	
Spontaneous premature	7 (6.5)
Perimenopause	29 (27.1)
Menopause from >3 years	55 (51.4)
Menopause from >6 years	10 (9.3)
Surgical menopause	6 (5.6%)
Treatment	
Cimicifuga racemosa herbal extract titrated in 2.5% of actein	
500 mg daily	49 (45.8)
500 mg + other plants	33 (30.9)
1000 mg daily	11 (10.3)
1000 mg + other plants	14 (13)
Clinical history	
Depression or anxiety	89 (83.1)
Moderate alcohol consumption	86 (80.3)
Chronic headaches	27 (25.2)
Dyspepsia	16 (15)
Hypertension	12 (11.2)
Thyroid disease	8 (7.4)
Hepatobiliary disease	5 (4.6)
Allergies	5 (4.6)
Previous cancer non genital organ	3 (2.8)
Major heart disease	2 (1.8)
Autoimmunity disease	1 (0.93)
Major psychiatric diseases	0
Alcohol abuse	0
Previous idiosyncratic reactions to drugs	0
Concomitant medication	
Antacids	26 (24.3)
NSAID	25 (23.3)
HRT	17 (15.9)
Hypotensive	12 (11.2)
Hormone thyroid therapy	8 (7.5)
Botanicals	5 (4.6)
Administration of blood product	0
Antiestrogen drugs	0
Vitamins	0

**Table 2:** Follow up of patients assuming *Cimicifuga.*

Parameter	Values
Physical examination	
BMI	28.5 (SD 0.71)
Blood pressure	
systolic	125 (SD 7.07)
diastolic	75 (SD 21.21)
Heart rate	76.5 (SD 0.71)
Few patients (9/107) were suffering of minor complaints, that revalued after 1 month did not show any sign of liver disease	
Abdominal pain	5 (4.6%)
Paresthesias	3 (2.8%)
Fatigue	2 (1.8%)
Anorexia	1 (0.9%)
Myalgia	1 (0.9%)
Malaise	1 (0.9%)
Pruritus	1 (0.9%)
Fever	0
Skin rash	0
Jaundice	0
Laboratory findings	
Leukocyte total count	4250 mm^3^ (SD 1060)
Bilirubin	0.75 mg dl^−1^ (SD 0.07)
AST	29.5 IU^−1^ (SD 10.61)
ALT	15.5 IU l^−1^ (SD 17.68)
Alkaline Phosphatase	135 U l^−1^ (SD 63.64)
GGT	28 U l^−1^ (SD 7.07)
Albumin	3.75 g dl^−1^ (SD 0.49)
INR	1 (SD 0.14)

**Table 3:** Follow up of five patients suffering from stable chronic liver disease.

Patients at baseline and control	1 Baseline	1 Control	2 Baseline	2 Control	3 Baseline	3 Control	4 Baseline	4 Control	5 Baseline	5 Control
Leukocyte	5780 mm^3^	6300 mm^3^	3990 mm^3^	4100 mm^3^	6700 mm^3^	6900 mm^3^	8000 mm^3^	7450 mm^3^	3450 mm^3^	4500 mm^3^
Tot bilirubin	1.4 mg dl^−1^	1.3 mg dl^−1^	0.9 mg dl^−1^	0.9 mg dl^−1^	0.7 mg dl^−1^	0.6 mg dl^−1^	0.2 mg dl^−1^	0.3 mg dl^−1^	0.4 mg dl^−1^	0.5 mg dl^−1^
AST	57 IU l^−1^	67 IU l^−1^	38 IU l^−1^	39 IU l^−1^	45 IU l^−1^	46 IU l^−1^	38 IU l^−1^	42 IU l^−1^	41 IU l^−1^	33 IU l^−1^
ALT	47 IU l^−1^	52 IU l^−1^	36 IU l^−1^	33 IU l^−1^	37 IU l^−1^	35 IU l^−1^	20 IU l^−1^	18 IU l^−1^	45 IU l^−1^	42 IU l^−1^
Alkaline phosphatase	150 U l^−1^	156 U l^−1^	119 U l^−1^	118 U l^−1^	110 U l^−1^	105 U l^−1^	120 U l^−1^	122 U l^−1^	110 U l^−1^	80 U l^−1^
*γ*GT	55 U l^−1^	59 U l^−1^	22 U l^−1^	25 U l^−1^	35 U l^−1^	32 U l^−1^	45 U l^−1^	44 U l^−1^	48 U l^−1^	35 U l^−1^
Albumin	3.5 g dl^−1^	3.6 g dl^−1^	4.1 g dl^−1^	4.2 g dl^−1^	3 g dl^−1^	2.9 g dl^−1^	3.8 g dl^−1^	4 g dl^−1^	4.2 g dl^−1^	4.4 g dl^−1^
INR	0.8	0.9	1.2	1.3	0.9	0.9	1.1	1.1	1	1.2
